# Dynamics, nanomechanics and signal transduction in reelin repeats

**DOI:** 10.1038/s41598-019-55461-8

**Published:** 2019-12-12

**Authors:** Karolina Mikulska-Ruminska, Janusz Strzelecki, Wieslaw Nowak

**Affiliations:** Institute of Physics, Faculty of Physics, Astronomy and Informatics, Nicolaus Copernicus University, Grudziadzka 5, 87-100 Torun, Poland

**Keywords:** Computational biophysics, Single-molecule biophysics

## Abstract

Reelin is a large glycoprotein controlling brain development and cell adhesion. It regulates the positioning of neurons, as well as neurotransmission and memory formation. Perturbations in reelin signaling are linked to psychiatric disorders. Reelin participates in signal transduction by binding to the lipoprotein receptors VLDLR and ApoER2 through its central region. This part is rich in repeating BNR-EGF-BNR modules. We used standard molecular dynamics, steered molecular dynamics, and perturbation response scanning computational methods to characterize unique dynamical properties of reelin modules involved in signaling. Each module has specific sensors and effectors arranged in a similar topology. In the modules studied, disulfide bridges play a protective role, probably making both selective binding and protease activity of reelin possible. Results of single reelin molecule stretching by atomic force microscopy provide the first data on the mechanical stability of individual reelin domains. The forces required for partial unfolding of the modules studied are below 60 pN.

## Introduction

Proteins control signal transduction between cells^1^. Defects in their sequence, structure, folding, dynamics or interactions may result in neurological diseases such as autism spectrum disorders (ASD), schizophrenia, bipolar disorder, depression or Alzheimer’s disease^2–4^. A murine mutant line called *reeler* was reported several decades ago^5^ and had a profound impact on the understanding of mammalian brain development mechanisms. In the *reeler* phenotype mice, the *Reln* gene is missing^6^ and therefore the process of neuron migration and brain architecture are compromised. This knockout results in an inverted cerebral cortex^7^. *The Reln* gene, located on chromosome 7 in humans, encodes reelin (RELN) - a large (385 kDa) signal glycoprotein localized in the extracellular matrix. The number of residues in RELN varies from 3008 amino acids (aa) in cattle to 3472 aa in a dog sequence. The loss of RELN in humans causes lissencephaly^8^.

The physiological roles of RELN are numerous and new ones are still being discovered^9–11^. RELN is expressed not only in the developing brain but also in the retina, liver, thyroid gland, adrenal gland, fallopian tube, and breast. It has been found even in the cornea and retinal ganglion cells^12^. This protein may also be involved in the relationship between dental nerves and odontoblasts^13^ participating in pain transduction^14^. RELN promotes dendrite growth and plays a role in long-term potentiation and synaptic plasticity in the adult hippocampus^15^. The Reelin and Notch signaling pathways are coupled and their presence in radial glia is important for spinal motor neuron localization as shown by Lee and Song^16^. Moreover, Matsunga *et al*. have indicated that RELN directly promotes N-cadherin-dependent neuronal adhesion, causing neuronal aggregation^17^. Thus RELN, through relaying biological signals, is a major player in brain development and functioning^18^. It also has numerous functions outside neuronal tissues: there is a mounting evidence that reelin signaling mechanisms may promote migration of cancer cells^11^.

RELN participates in canonical signaling by binding to the very low-density lipoprotein receptor (VLDLR) and apolipoprotein E receptor 2 (ApoER2)^17^. This event induces tyrosine phosphorylation of the adaptor protein disabled 1 (Dab1), a process mediated by Src kinases. Phosphorylated Dab1 activates in turn downstream signaling cascades, including the PI3-kinase-dependent pathway^10^. The structures of reelin receptors are only partially known^19^ and were discussed recently by Dlugosz and Nimpf^20^. Non-canonical RELN signaling pathways were postulated as well^21^.

In RELN, several regions are distinguished: a signal peptide, an F-spondin-like domain, eight reelin repeats (BEB1-8), and a positively charged sequence at the C-terminus. The 3-D structure of full-length RELN is not known yet, but central part (BEB3 and BEB5-6) has been determined using X-ray crystallography^22,23^. The main signaling events are based on binding of these central RELN modules to the receptors. Each BEB module has a characteristic structure and contains an epidermal growth factor (EGF) motif flanked by two bacterial neuraminidase repeats (BNR)^21^. The EGF and BNR domains are also present in other proteins, such as spondin-1. They contain up to 7 cysteine bridges and have rich topologies, including lasso motives^24^. Moreover, the fragment of RELN studied here contains two Zn^2+^ ions and a characteristic amino acid sequence, which correlates with the reported proteolytic function of reelin^25,26^.

The extracellular part of RELN is subject to mechanical stress during neural growth and tissue remodeling. The signaling may also be dependent on the specific rigidity of the protein or particular modules. Despite the high interest in RELN, neither the nanomechanics nor the dynamics of this molecule have been studied so far. Thus, we employed atomic force microscope (AFM) single molecule force spectroscopy (FS) in conjunction with steered molecular dynamics (SMD) simulations to characterize the mechanical unfolding and stability of critical fragments of RELN. From the results of classical MD simulations, we analyzed flexibility of various BEB modules. Finally, we computationally indicated sensor and effector regions, critical for RELN-receptor interaction, using the perturbation response scanning (PRS) method. The data provided by our study should provide a better understanding of reelin signaling pathways. This modeling enables us to determine the roles of mutations linked with neuronal diseases and brings new clues on mechanical control of RELN proteolytic activity.

## Materials and Methods

**Figure 1:**
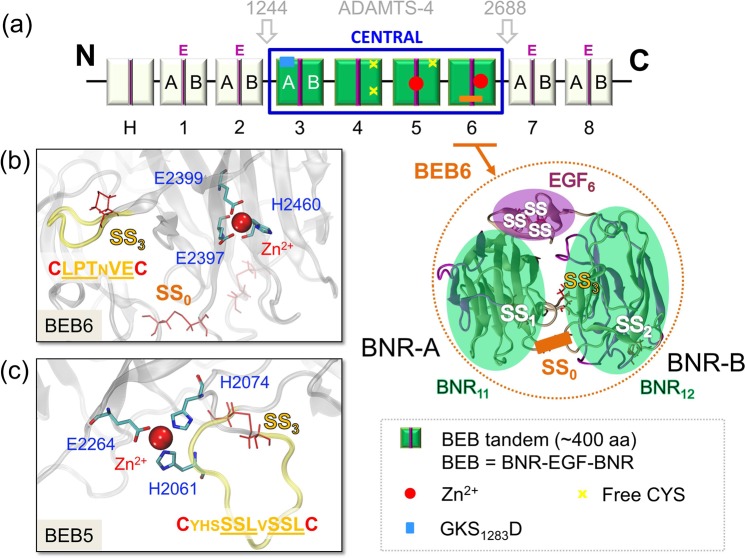
Multidomain structure of RELN. (**a**) Full structure of RELN in a schematic, multidomain representation and its proteolytic fragments after N- and C-terminal processing by ADAMTS-4 (P1244-A2688) are displayed. *Green rectangles* represent BNR domains, and EGF domains are colored in *dark pink*. RELN contains eight subunits, which consist of BNR-EGF-BNR domains (BEB). The *blue box* denotes the central part of RELN that was studied in the paper. *Red dots* show Zn^2+^ binding sites and *yellow crosses* represent cysteines that do not form a disulfide bond. C2101 located in BEB5 is responsible for RELN homodimerization. The enzymatically decisive serine residue in the GKS_1283_D sequence is indicated by a *blue* rectangle. The crystal structure in the inset shows the 3-D structure shared by BEB modules with all possible disulfide bonds displayed: (i) disulfide bonds within an EGF domain (named SS), (ii) disulfide bonds that zip BNR domains (SS_1_ in subunit BNR-A and SS_2_ in subunit BNR-B), (iii) a disulfide bond that keeps together a loop comprised of residues 8–12 (SS_3_), and (iv) a disulfide bond that links BNR11 with BNR12 (SS_0_) which is characteristic to BEB6 only. Close views of Zn^2+^ binding sites in (**b**) BEB6 and (**c**) BEB5 are displayed in the insets. Residues which coordinate Zn^2+^ are shown: H2061, H2074, E2264 (in BEB5) and E2397, E2399, H2460 (in BEB6). The *yellow* fragment of the protein (whose sequence is displayed) corresponds to the loop located in vicinity of the Zn^2+^ binding site and protected by SS_3_ in both BEB5 and BEB6 modules. The SS_0_ bond (*orange*) is also displayed in BEB6.

Mouse RELN used in this study contains 3461 amino acids and consists of a 27-residue long signal peptide, segment ‘H’ and 8 characteristic BEB repeats^2^. Each repeat contains an EGF motif at the center, flanked by two BNR repeats, A (left BNR) and B (right BNR) (Fig. 1).

The most significant region of RELN is located in the middle part of the full sequence. That region was shown to be crucial for interactions with other proteins^27^. Lack of even a small part of the BEB3-BEB6 fragment caused the dissociation of the complex with its partners ApoER2 and VLDLR^27^. The BEB3-BEB6 fragment is considered as the most significant part of RELN so we investigated this system experimentally and computationally.

### Molecular dynamics (MD) simulations

RELN is a large protein composed of 16 BNR and 8 EGF domains. Its full structure has not been solved yet. In our modeling we included four of the most important mouse RELN fragments: BNR5-EGF3-BNR6, BNR7-EGF4-BNR8, BNR9-EGF5-BNR10 and BNR11-EGF6-BNR12; called respectively: BEB3, BEB4, BEB5 and BEB6 (see Table 1, Fig. 1). The structures of BEB3 and BEB5-6 were retrieved from the Protein Data Bank^28^ whereas BEB4 module, absent in the PDB, was built by the Swiss Model^29,30^ server based on high homology (34% sequence identity) with a similar fragment, i.e. the 2ddu model of the BEB3 structure. Except standard amino acids, each BEB structure consists of two Ca^2+^ ions, several sugars attached to the protein backbone (such as N-acetyl-D-glucosamines (NAGs) and beta-D-mannose (BMA)), and bound waters. All these elements as well as Zn^2+^ ions present in BEB5-6, were included in the modeled systems.

**Table 1:** Reelin fragments used in the all-atom MD and SMD simulations.

Structure	Domains	PDB code	Residue range	No of atoms
BEB3	BNR5-EGF3-BNR6	2ddu^23^	T1235-L1600	32 109
BEB4	BNR7-EGF4-BNR8	model	S1603-I1947	29 942
BEB5	BNR9-EGF5-BNR10	2e26^22^	V1956-N2317	35 098
BEB6	BNR11-EGF6-BNR12	2e26^22^	T2322-S2662	29 317

Mouse RELN has 99.2% sequence similarity to the human protein. The NAMD^31^ code with the CHARMM27 force field^32^ was used to perform all-atom MD and SMD^33^ simulations. In all preparatory simulations, explicit water molecules (TIP3P) were used and the following protocol was adopted: (1) 0.2 ns of water equilibration, (2) 10,000 steps of minimization, (3) 0.35 ns of heating from 0 to 300 K, (4) 0.15 ns equilibration of the whole system. For each structure five MD trajectories were generated, each 100 ns long with a 2-fs time step. A cutoff of 12 Å for non-bonded interactions was applied. Langevin dynamics and the Langevin piston algorithm were used to maintain temperature at 300 K and pressure at 1 atm. We used VMD^34^ for visualization and the ProDy^35^ API with in-house prepared codes for the data analysis. The first 10 ns of all MD trajectories were omitted during the analysis.

**Figure 2:**
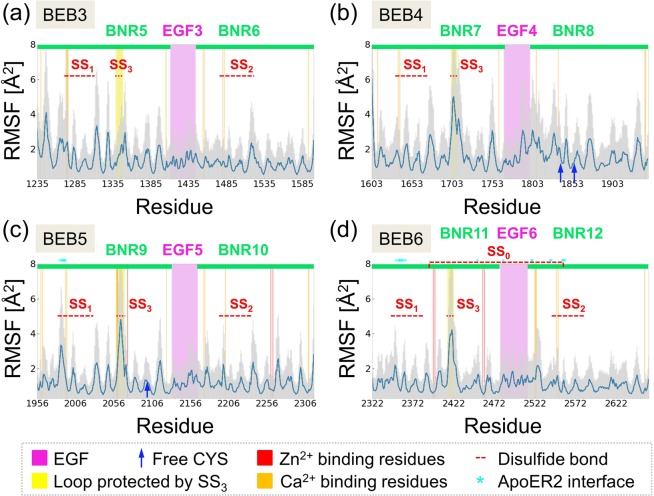
RMSF results for BEB3-BEB6 from MD simulations. Fluctuations include: (**a**) BEB3, (**b**) BEB4, (**c**) BEB5 and (**d**) BEB6. BNR domains are denoted by *green bars* and the regions that belong to EGF domains are in *pink*. *Yellow bars* highlight loops that are protected by an SS_3_ type disulfide bond, which is common for all BEBs in RELN. *Red* and *orange* sites are residues within 3.5 Å from the Zn^2+^ and Ca^2+^ ions, respectively (a list is in Table S1). *Red dotted lines* along the abscissa highlight regions that are connected by disulfide bonds and *blue arrows* point to sites containing a cysteine which does not create any disulfide bond. Residues that interact with ApoER2 receptors within 4 Å radius are indicated by *cyan stars* (based on PDB ID 5b4x, data in Table S1).

To check convergence of MD simulations, we have performed all analysis for sets of two 100 ns trajectories, and then compared the results with data obtained from sets of five 100 ns trajectories. Doubling of the simulation time did not affect any structural observations and conclusions. The average RMSF fluctuations were taken for further analysis, the statistical error bars are indicated as shadows in Fig. 2. The same convergence check was performed for calculated correlation matrices. Only minute changes in spans of the correlated-anti-correlated regions were noticed after substantial extensions of the conformational spaces sampled from two to five 100 ns for each system.

### Steered molecular dynamics simulations

To check the stability and mechanical unfolding processes of BEB structures, six SMD simulations were performed for each of the BEB3-BEB5 systems and three for BEB6. The protocol was the same as in the classical MD described above except that the constant velocity SMD scheme was used to stretch each BEB structure along its N-to-C vector at a constant speed of 0.025 Å/ps. In the SMD simulations, the C_α_ atoms of the C-terminus of each BEB structure were fixed whereas an external harmonic force with a spring constant of 278 pN/Å was applied to C_α_ atoms from the N-terminus.

Each unfolding trajectory is slightly different, especially in the higher extension or force regimes, but all six scenarios are very consistent. The maximum error in SMD elongation values is 10 nm, so it does not exceed 10% of the calculated values. One should note that SMD forces strongly depend on the pulling speed. Rates of pulling used in simulations, for practical purposes, are orders of magnitude higher than those in AFM experiments, so the calculated values may be only qualitatively correlated with real AFM force spectra.

### Elastic network model and perturbation response scanning analysis

Our study included Elastic Network Model (ENM)-based analysis such as Perturbation Response Scanning (PRS)^36,37^. PRS provides maps in which the *ij*^*th*^ element describes the effect of perturbing one residue *i* on the dynamics of another residue *j*. Each row corresponds to the perturbation of a given residue *i*, and the elements of the row show the response of all other residues (receivers) in a protein. The average effect of all perturbed residues of each row is called effectiveness and indicates the ability of residue *i* to affect dynamics changes in all other residues. Thus, the strongest effectors can be interpreted as elements that apply global control over propagation of allosteric signals to a protein partner^38^. In turn, the *j*^*th*^ column represents the level of perturbation for the residue *j* to the perturbations of every single residue in the protein structure. The strongest sensors can be interpreted as effective receivers of allosteric signals. The sensors are freely accessible and therefore can be involved in the execution of allosteric structural changes^38^. PRS has been successfully used for other biological systems^39–41^. The ProDy API^35^ was used, together with in-house prepared codes, for the PRS analysis.

### Sequence analysis and generation of sequence-based cladograms

Sequence analysis and comparisons were performed using as a metric the pairwise Hamming distances. Sequences were obtained from the UniProt database^42^. A dendrogram was generated by in-house code and the ProDy API^35^, and visualized using the iTOL server^43^.

### Single molecule force spectroscopy

An atomic Force Microscope (AFM) in the force spectroscopy mode was used^44–47^. Experiments were made on mouse reelin samples, obtained from R&D Systems (3820-MR-025). The samples contained only the BEB3-BEB6 fragment of the protein. The primary sample was reconstituted in PBS buffer (Sigma-Aldrich 4417) to 100 µg/ml concentration. In order to minimize RELN aggregation^48^, the sample was put in a vortex for 30 minutes prior to each experiment, diluted to 10 µg/ml, and shaken again. Such solutions were incubated on a gold-coated glass substrate (Arrandee 11 × 11 mm) for six hours. Afterwards the unbound molecules were washed away with PBS buffer. The AFM measurements were performed with a custom-made AFM one-dimensional puller, described in detail by Pawlak and Strzelecki^49^. Bruker MLCT-C cantilevers with a nominal spring constant of 10 mN/m were used. Each cantilever was calibrated prior to each experiment using the thermal tune method^50^. The experiment involved repeatable approaching of the AFM cantilever tip to the protein-covered substrate in order to attach single RELN molecules with nonspecific binding. To increase the probability of selective binding of single molecules only, no direct tip-surface contact was made. This approach known as “fly fishing”^51^ was successfully used for other systems^44,46,47^. The collected force curves were analyzed with in-lab developed software. Our program allows for force spectra fitting with a Worm Like Chain^52^ (WLC) model of biopolymers. As RELN aggregation was noticed in all initial experiments, the incubation time was decreased to two hours and the protein concentration was lowered to 5 µg/ml. The accuracy of the force determination was 8.5 pN, estimated based of thermal noise inherent to MLCT-C cantilevers. The lengths were measured with an error of 0.15 nm, imposed by the piezo stage.

## Results and Discussion

### Stability of reelin modules – the role of disulfide bonds

Reelin repeats (BEBs) are particularly rich in disulfide bonds, which may selectively stabilize individual modules (Fig. 1). To compare the thermal stability of different BEBs, we performed classical MD simulations and computed root mean square fluctuations (RMSF values) for each residue. Data are presented in Fig. 2. Specific regions are color-coded: BNR (*green bar*), EGF (*pink box*), and residues that form disulfide bonds (*red dotted lines*). Each BEB module contains from five to seven disulfide bridges while three bonds are present in a typical EGF domain (Fig. 1a, denoted SS).

A characteristic disulfide bond localized in a BNR domain keeps together a loop, which consists of eight to twelve residues (Fig. 1, SS_3_ and Fig. 2, *yellow boxes*). In BEB5-BEB6 modules those characteristic loops are localized close to the Zn^2+^ binding site, which is localized on the opposite site to the ApoER2 binding interface (PDB ID: 5b4x). The other two disulfide bridges zip the structure of BNRs and preserve the spatial arrangement of the calcium binding site (Fig. 1a, SS_1_/SS_2_ and Fig. 2, *orange vertical lines*). In the RELN structure considered in this work there are two exceptions from this general scheme: one is the BEB4 structure, which does not form a disulfide bond in the BNR8 (BNR-B) domain, and the second is BEB6 that has one extra disulfide bond between the BNR11 and BNR12 modules (Fig. 2d, SS_0_ and Fig. 1a). It remains unclear why these couplings are modified in comparison with the other BEBs.

We have analyzed loops present in the central part of RELN using the LassoProt server^24^. The simplest type of loops (L_0_) is localized in EGF domains (three L_0_ loops) and in BNR domains (*yellow box*, Fig. 2). More complex loops such as L_−1C_, containing a single lasso with a covalent loop pierced once by the C tail, has been detected in all BNR domains (C1271-C1310, C1475-C1522, C1633-C1673, C1983-C2030, C2195-C2235, C2348-C2387, C2544-C2584; *red dotted lines* in Fig. 2) except for BNR8. The same class of loops but with N tail piercing, labeled as L_−1N_, is present between C2393 and C2559. It has the N-terminal crossing at Q2380, which is characteristic for BEB6 module.

Fluctuation profiles (Fig. 2) of all BEBs are similar; the accuracy of these data may be estimated from the plots of standard deviations based on five independent trajectories (Fig. 2). Two different dynamics profiles of EGF domains were found. The first group is characterized by limited mobility where the maximum value of RMSF does not exceed 2 Å^2^. Such stiff regions are present in BEB3, BEB5 and BEB6 (Fig. 2a,c,d, *pink box*). The second group has a more mobile region at the C-terminus of the EGF domain where the RMSF value reaches the level of 4 Å^2^. Such a profile is noted in BEB4 (Fig. 2b, *pink*), in which SS_3_ bond is absent. Notably, the EGF region in BEB5 belongs to the binding site interface of the receptor ApoER2. This binding interface region is specified in Table S1. Elevated fluctuations of ~4–5 Å^2^ can be observed for a loop protected by the SS_3_ bond (Fig. 2b–d, *yellow box*), which is localized close to the Zn^2+^ binding site (see Fig. 1c). This site may play a catalytic function since three residues that coordinate the Zn^2+^ ion in BEB5 (H2061, H2074, E2264) are common for a typical catalytic zinc site.

One should note that the whole region of BEB3-BEB6 is crucial for successful binding of ApoER2 and VLDLR since the lack of even a small part of BEB3-BEB6 leads to the unbinding of both RELN receptors^27^. Similarities in the RMSF profiles between BEB3, BEB5 and BEB6 suggest that the SS_0_ bond, present in BEB6 only, does not have any special impact on the mobility of individual BEB6 residues. Thus, we infer that its function is to prevent the BNR11-BNR12 modules’ disconnection during binding to the RELN receptors. This hypothesis should be further investigated using mutagenesis studies.

In contrast to the other BEB modules of RELN, BEB4 has a smaller number of S-S bridges. In order to estimate what effect an internal disulfide bond has on BEB dynamics we performed auxiliary simulations of BEB3 having the S-S bridge in BNR6 domain (BNR-B) cut. Interestingly, the RMSF values of the modified BEB3 show that the absence of this disulfide bond increases the level of fluctuation in the BNR5 domain and may affect the region close to the calcium binding site in this BNR-A domain (see Fig. S1). Moreover, lack of this S-S bridge affects dynamics of the area around the residue ~G1350 located in close proximity to the SS_3_ loop protected region (Fig. 2a, *yellow box*). Such long-distance effects of the S-S bridge absence/presence in BEB modules are also supported by changes in the cross-correlations (see Fig. S2).

### Correlations in reelin modules

**Figure 3:**
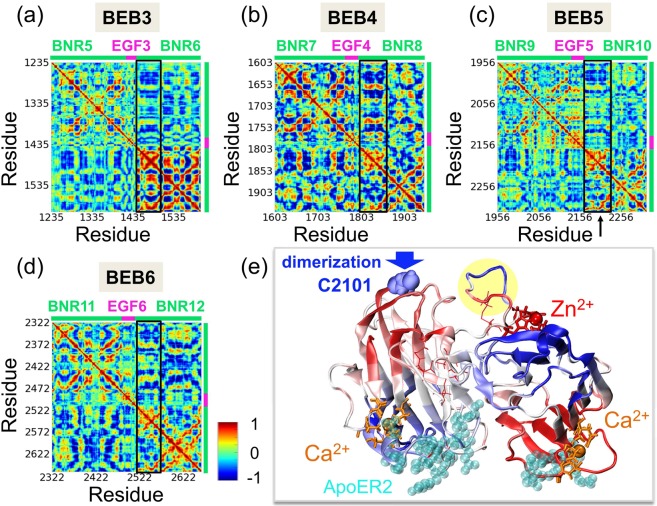
Cross-correlation of BEBs structures in MD simulations. Cross-correlation heatmaps for individual BEB structures: (**a**) BEB3, (**b**) BEB4, (**c**) BEB5 and (**d**) BEB6 show which regions tend to move in the same (correlated, colored in *red*) or in the opposite (anti-correlated, colored in *blue*) directions with respect to each other in the global modes. (**e**) The crystal structure of BEB5 fragment colored by values obtained from cross-correlation analysis for the residue K2194 which is located in the *black box* region (a *black arrow* denotes that residue). Ca^2+^ interface residues are displayed as *orange sticks*, those for Zn^2+^ in *red sticks*, and ApoER2 binding site is colored in *cyan*. A loop protected by SS_3_ (displayed in Fig. 1c) is denoted by a *yellow shadow*. Disulfide bonds are shown as *thin red sticks* and C2101, which serves for RELN dimerization, is denoted by *blue spheres*.

Pairwise cross-correlation matrices of BEB3-BEB6 structures, based on the average of the ten slowest modes from analysis of MD simulations, are shown in Fig. 3. The matrices provide information on correlations between motions of different BEB regions. The analysis reveals which regions of the protein tend to move in the same (correlated) or in opposite (anti-correlated) relative directions in the global modes. The correlation values are normalized by the mean square fluctuations and vary from −1 (strong anti-correlation; *dark blue*) to 1 (strong positive correlation; *dark red*). The bar on the top of the maps shown in Fig. 3 corresponds to the two BNR domains linked by EGF module as denoted, respectively, by *green*, *pink and green colors*. The upper-left and lower-right quadrants correspond to the BNR domain correlations, and the center region illustrates the EGF domain correlations with respect to the other elements of BEB modules.

The main similarity in the internal dynamics of BEB structures (Fig. 3) is found in the first half of BNR-B domains, which tend to be strongly correlated (note the *red stripes*). This region belongs to the calcium binding site and implies both a high correlation (Fig. 3a–d, *black boxes, dark red*) and an anti-correlation (Fig. 3a–d, *black boxes, dark blue*) with respect to a part of the BNR-A module. This feature is convergent, i.e. it is stable with respect to increasing number of trajectories used in correlation matrix calculations. Moreover, it seems to be common in all BEB structures studied and is not very affected by the lack or presence of stabilizing disulfide bonds. However, these regions start to be even more anti-correlated after deleting the disulfide bond in the BNR-B domain. We draw this observation from additional test calculations for a modified BEB3 structure (see Fig. S2).

Thus, for a better understanding of this general internal dynamics feature of reelin modules, we display cross-correlation values of a selected region mapped on the structure of BEB5 (Fig. 3e, *a black arrow* indicates the region providing data). We note that the ApoER2 binding interface in BNR-A is coupled (both *dark red*) with the BNR-A calcium binding site and BNR-B zinc binding area. Moreover, this interface is also anti-correlated (therefore also coupled) with the calcium binding residues in BNR-B (*dark blue*). These results of MD modelling indicate that possible mechanical strains in the ApoER2 binding site may not only affect the Ca^2+^ interface significantly, but also perturb the postulated Zn^2+^ catalytic site located on the opposite side of the BEB reelin module.

In the central part of all maps we have a quadrant corresponding to the cross-correlations of an EGF domain. The EFG domains are uncorrelated with BNR-A type domains (we see mainly *green* colors, i.e. cross-correlation values around 0) but highly anti-correlated with some regions of BNR-B modules.

### Signal relay in RELN

Next, we examined possible pathways of allosteric signaling in RELN using the PRS approach, which provides the magnitude and directionality of the residue displacements in response to an external force. This approach is based on linear response theory and it has been successfully used for revealing elements of a protein structure crucial for allosteric signal transduction^37–39^.

**Figure 4:**
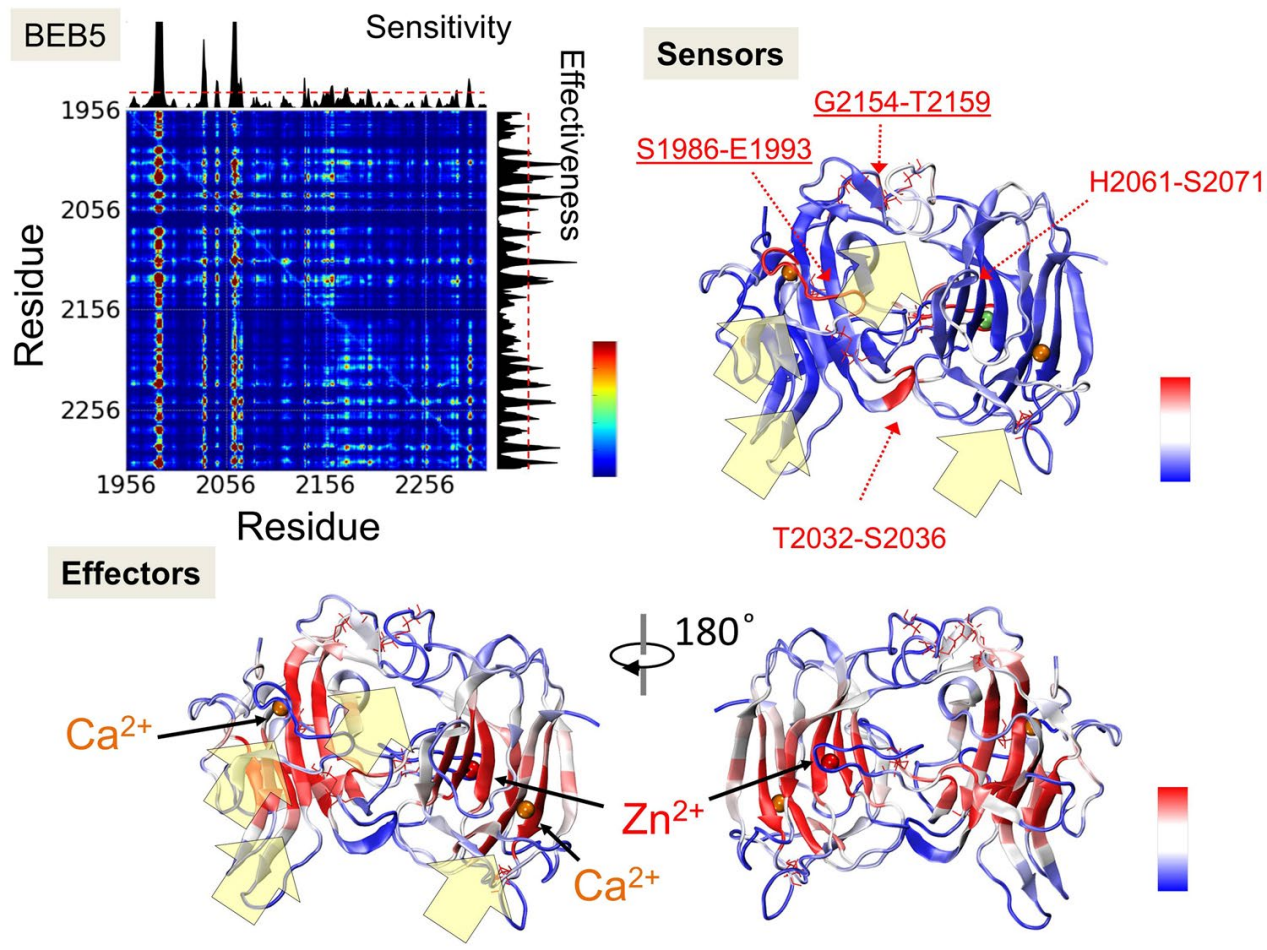
PRS analysis of BEB5 module. The PRS map illustrates the strongest perturbation (in *dark red*), which describes the effect of perturbing a residue *i* on the dynamics of a residue *j*. Sensitivity and effectiveness profiles are shown along the top and right sides of the map. The highest values on a sensitivity profile correspond to the strongest sensors and the highest values of effectiveness correspond to the strongest effectors. Both profiles are superimposed on the crystal structure of BEB5. Ca^2+^ and Zn^2+^ ions are denoted by *orange* and *red* spheres, respectively. Except, for clear visualization on the sensitivity profile, the Zn^2+^ ion is denoted as a *green sphere*. *Yellow* arrows show the binding sites of ApoER2.

**Figure 5:**
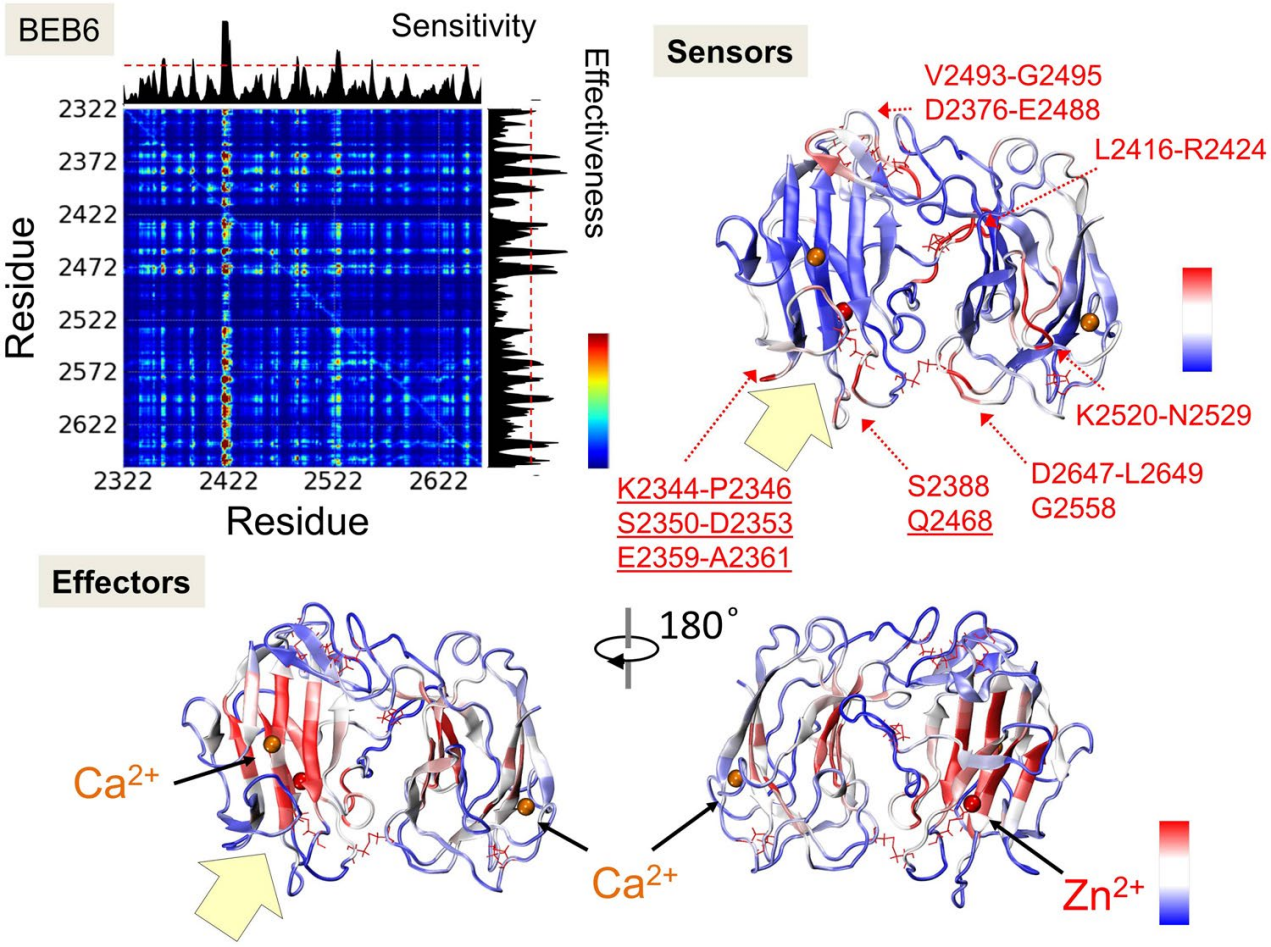
PRS analysis of BEB6 module. The PRS map illustrates the strongest perturbation (in *dark red*), which describes the effect of perturbing residue *i* on the dynamics of residue *j*. Sensitivity and effectiveness profiles are shown along the top and right sides. The highest values on a sensitivity profile correspond to the strongest sensors and the highest values of effectiveness correspond to the strongest effectors. Both profiles are displayed on the crystal structure of BEB5. Ca^2+^ and Zn^2+^ ions are denoted by *orange* and *red* spheres, respectively. *Yellow* arrows show the binding sites of ApoER2.

In Figs. 4 and 5, we present PRS maps calculated for BEB5 and BEB6 structures. In PRS maps, the *ij*^*th*^ element describes the effect of perturbing a residue *i* on the dynamics of a residue *j*. Thus, it evaluates the strongest sensors and effectors of each structure. The strongest sensors and effectors are denoted in the crystal structure scheme of RELN in *dark red* whereas residues which do not exhibit those properties are colored in *dark blue*. The analysis shows that effectors (residues which propagate an allosteric signal) are buried inside BNR domains whereas residues denoted as potential receivers of allosteric signals (strong sensors) were identified in regions that are freely accessible to other molecules i.e. (i) the binding interface of ApoER2 (Figs. 4, 5, underlined residues and *yellow arrows*), (ii) ion binding sites (Zn^2+^ or Ca^2+^) and (iii) a fragment of the EGF domain. The results clearly imply the presence of a signal transduction pathway between Zn^2+^ and ApoER2/Ca^2+^ binding sites in both modules, BEB5 and BEB6. It may suggest an enzymatic function of RELN, for example, as a serine protease^53^, and the importance of ApoER2/Ca^2+^ impact on the Zn^2+^ binding site already noticed in the cross-correlation analysis. The zinc binding site in BEB5 (see Fig. 1c), coordinated by H2061, H2074 and E2264, creates a common form of a catalytic site for which we hypothesize that H2061 and S2062 residues may belong to the catalytic triad: (located in the SS_3_ protected loop region, see Fig. 1c, *yellow loop*). The zinc binding site in BEB6 is composed of three residues, i.e. E2399, E2397 and H2460 (Fig. 1b), which are also common for catalytic zinc settings. Interestingly, in BEB6 the SS_3_ protected loop is located significantly further (>20 Å) from the zinc ion than in the BEB5 module. Nevertheless, we are rather convinced that this loop plays an important role in RELN functionality since the motif observed here, namely LPTxVE (x being any residue, Fig. 1b) is present in >220 protein sequences and most of them have an enzymatic function. This information was obtained by the *MOTIF search tool*^54^.

The PRS calculations were performed for BEB3 and BEB4 modules as well (see Figs. S3 and S4). Unlike BEB5 and BEB6, they do not contain any zinc catalytic sites, nevertheless they still share similarities in localization of effectors. Effectors are here buried in BNR domains. In BEB3 and BEB4, we identified sensors localized in loops near the calcium binding site region, in BNR5 and BNR8, but not in BNR6 or BNR7. Moreover, there are strong sensors in BEB3 and BEB4, localized in the lower part of BNR-A domains which includes residues K1282-G1285, Q1313-T1317 and S1395-Q1396 in BNR5 (a part of BEB3), and T1752-A1755 and K1675-S1678 in BNR7 (a part of BEB4). The possible function of those regions in BEB4 remains unknown. Our strong BEB3 sensor GKS_1283_D, has been recognized as an enzymatically active center at position S1283^25^. Likewise, sensors are also present in the lower part of BNR-B domains: K1585-H1586, K1516-T1521 in BNR6 (a part of BEB3) and R1826-T1833, N1933-E1938 in BNR8 (a part of BEB4).

Summarizing this section, we postulate that the PRS analysis gives useful hints regarding possible pathways of interaction signal transduction within characteristic reelin motifs (BEB) studied here. These data may now be critically compared with dynamics of the numerous other protein fragments exhibiting the same BEB type fold.

### Conservation of RELN sequence

**Figure 6:**
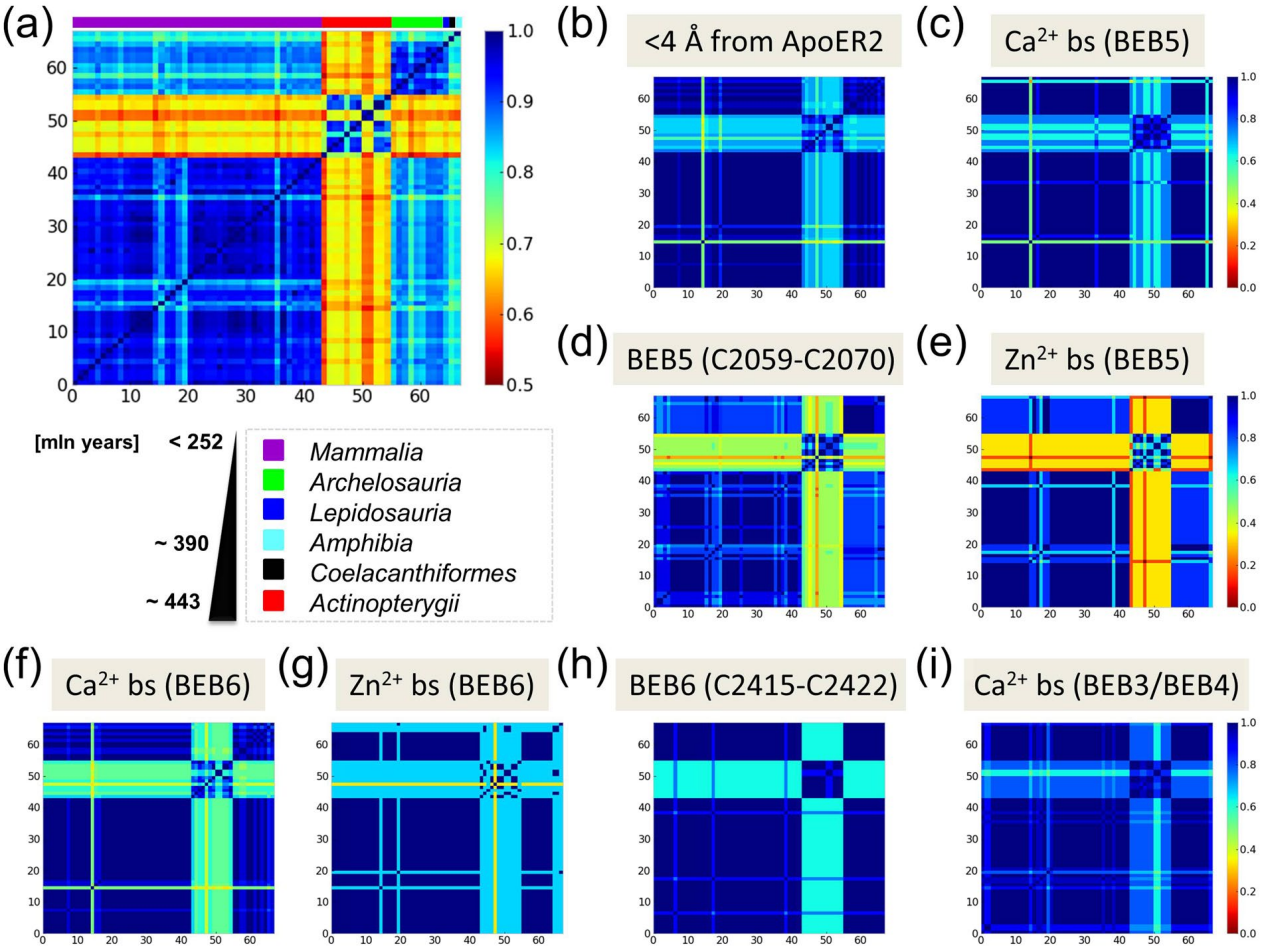
Distance matrices for RELN sequence conservation estimates. (**a**) Conservation of RELN sequence based on the Shannon entropy measure. Color code of bars on the top of the heatmap corresponds to the name of class assigned by a pySCA script (available under https://github.com/reynoldsk/pySCA) that uses Pfam sequence annotations. *Dark blue* color of the heatmap corresponds to a high conservation of residues. Heatmaps with conservations for: (**b**) ApoER2 binding site in BEB5 and BEB6 for residues within 4 Å, (**c**) calcium binding site (bs) in BEB5, (**d**) SS_3_ protected loop in BEB5 (C2059-C2070), (**e**) zinc binding site in BEB5, (**f**) calcium binding site in BEB6, (**g**) zinc binding site in BEB6, (**h**) SS_3_ protected loop in BEB6 (C2415-C2422), (**i**) calcium binding site in BEB3 and BEB4. All residues are enumerated in Table S1.

Phylogenetic analysis methods are widely used to estimate relationships among biological species to detect evolutionary information. To systematically compare the conservation level in RELN sequences and the importance of specific regions found in our PRS analysis, we selected 67 sequences of RELN from different organisms stored in the UniProt database^42^. The sequences consist of 6 classes (see Fig. 6a). The youngest sequences belong to mammals while the oldest belong to *Actinopterygii*. This last group seems to be quite different (40–60% lower conservation) in comparison to the other sequences analyzed.

In Fig. 6, we show the distance matrices calculated for selected fragments of RELN sequences. We focused on checking the level of conservation for regions that have been revealed using the PRS analysis: (i) ApoER2 binding interface in BEB5-6 modules (Fig. 6a); (ii) Ca^2+^ binding sites for BEB3-6 (Fig. 6c,f,i); (iii) Zn^2+^ binding interfaces in BEB5 and BEB6 (Fig. 6e,g); and (iv) a characteristic loop protected by the SS_3_ bond in BEB5 and BEB6 modules (Fig. 6d,h and the 3-D view of the structure in Fig. 1b,c). Residues selected for this comparison are shown in Table S1. The results show the similar pattern in all selected regions and therefore indicate relations between ApoER2 binding interface residues, calcium binding residues from all BEBs as well as the zinc binding site, and SS_3_ protected loops in BEB5-6 modules. The evolution analysis implies that the same relationship between effectors and sensors holds in homologous proteins as that implied by the PRS analysis in our RELN protein.

### SMD mechanical unfolding of reelin modules

**Figure 7:**
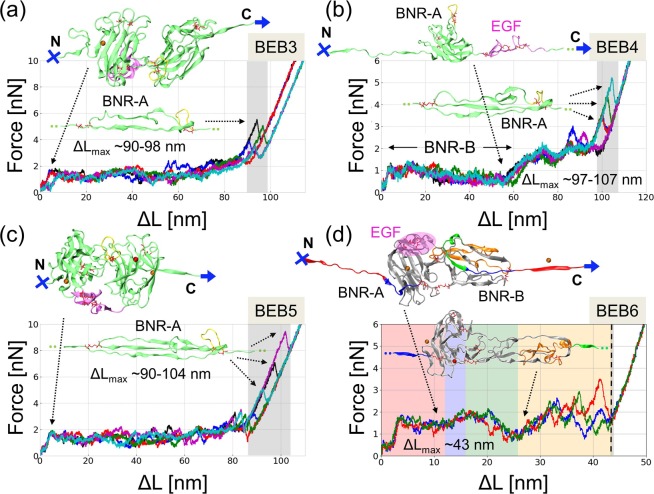
Unfolding of BEB3-6 modules in SMD simulations. Evolution of the force and the unfolding length for: (**a**) BEB3, (**b**) BEB4, (**c**) BEB5 and (**d**) BEB6. The accompanying ribbon diagrams show the snapshots from the simulations and characteristic events such as the unfolding blocked by the *yellow* loop protected by the SS_3_ bond (*panel a-c*, *lower ribbon diagram*). In *panels a-c*, BNR domains are colored in *green* and EGF domain in *magenta* while in *panel d*, colors denote the sequence of the unfolding events. Red sticks represent disulfide bonds, *red* spheres Zn^2+^ and *orange* sphere Ca^2+^ ions. A full elongation is reached at the ΔL_max_ value (a maximal N-C distance minus the initial N-C distance), which is ~90–98 nm for BEB3, ~97–107 nm for BEB4, 90–104 nm for BEB5, and 43 nm for BEB6 (*grey box*). In all upper ribbon diagrams N and C termini and the direction of pulling are shown. The divergence of forces and lengths are indicated.

RELN performs its function by forming a large protein complex with two transmembrane receptors, ApoER2 and VLDLR and therefore contributes to the mechanical stability of a cell^6^. One may compare multi-modular RELN to a spring connecting two objects and subject to mechanical tension, always present in developing tissue. Here we address the following questions: what is the effect of an external force applied to the central region of RELN? Which regions play a role of the buffer in RELN structure subject to tension? For this purpose, we used the steered MD (SMD) method to unfold each individual reelin BEB structure localized in the central part of the protein. Reelin modules’ unfolding scenarios are presented in Fig. 7.

The unfolding scenario of BEB3-5 modules, according to SMD results (Fig. 7), can be basically divided into four parts: I) a pre-burst state, extending from ΔL = 0 to around ΔL = 2-5 nm, during which the distance between two BNR subunits increases, but BEB still maintains its compact structure and the external force remains below 1000 pN (see Fig. 7a,c, *upper ribbon diagram*); II) a main burst state, when the secondary structure in BNR-B (or consecutively BNR-A and then BNR-B) begins to break down and the force reaches a value of ~2000 pN, (extension of ~5 nm to ~60 nm). Both these phases are common for BEB3/BEB5, but only phase I appears in BEB4 which does not contain the disulfide bond in the BNR-B domain (Fig. 7b, *upper ribbon diagram*). Therefore, the unfolding of the BEB4 module occurs under significantly lower forces than in other BNR-B domains (see Fig. 7b). III) a post-burst state completed, during extension from ~87–97 nm to ~110 nm; this part has a characteristic intermediate state induced by a slipknot created due to the presence of the SS_3_ disulfide bond which causes 8–12 residues to be zipped (Fig. 7a–c, *lower ribbon diagram* and Fig. 1b,c); IV) a fully elongated state, when the BEB polypeptide chain achieves its maximum length (*ΔL*_*max*_). The value of *ΔL*_*max*_ depends on the module: 90–98 nm for BEB3, 97–107 nm in BEB4 and 90–104 nm in BEB5. The variance of those values is associated with the hooking of the SS_3_ protected loop at the final stage of unfolding (Fig. 7a–c, *lower ribbon diagram*).

The BEB6 domain differs from the other BEBs due to the presence of an additional disulfide bond SS_0_ (Fig. 1a) keeping both BNR-A and B modules in close proximity. Therefore, except for the initial stage (an extension of 0–3.5 nm), the BEB6 unfolding scenario is unique. The BEB6 unfolding pathway is presented in Fig. 7d. Initially, despite the presence of SS_0,_ a large part of BNR-B domain unfolds in a way similar to other BEBs. However, when the unfolding length exceeds 26 nm (initial N-C terminal length is ~4.6 nm), the force starts to rise up to ~2500 pN and the second half of the BNR-B domain (Fig. 7d, *orange*) starts unfolding through the loop formed by the SS_0_ bond. A full elongation is reached after 43 nm and importantly, it does not affect the Zn^2+^ binding site of BEB6. In our modeling protocol extreme forces were applied in order to save computer time, but this result suggests that the presence of the SS_0_ bridge is important for RELN function. It may guard the relative orientation of the catalytic site residues coordinated to zinc, the SS_3_ protected loop (see Fig. 1b, *yellow*) and the EGF domain (Fig. 7d, *pink oval*).

### Single molecule AFM force spectroscopy measurements

AFM force spectroscopy experiments on BEB3-6 RELN fragment were challenging. The protein is prone to aggregation, which lowered the success rate for picking single molecules. Additionally, as observed previously^55^, BEB3-6 fragments tend to form dimers linking two monomers via Cys-Cys C2101 disulfide bonds in the BEB5 region. Thus, apart from often detected aggregated reelin, the AFM tip would pick and stretch a dimer of two BEB3-6 fragments instead of one.

**Figure 8:**
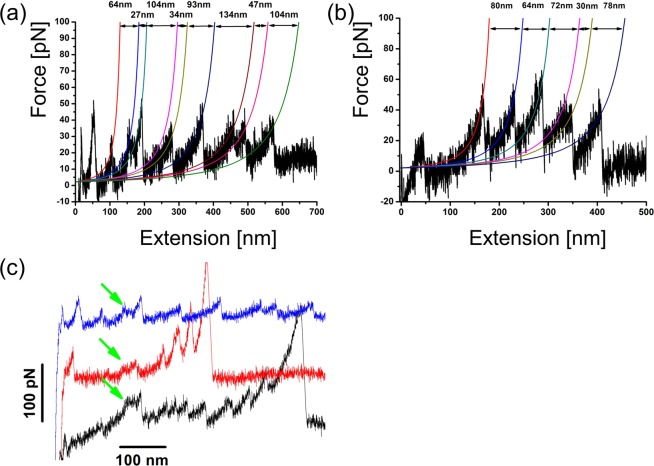
AFM force spectroscopy captures unfolding of individual BEB domains and pre-burst state extension (as predicted by SMD simulations). The representative curves obtained with reelin samples (**a,b**) show characteristic tooth patterns, corresponding to sequential unfolding of BEB repeats. Solid lines show WLC model fits to the data (with a persistence length of 0.4 nm). Calculated contour length increment values are also shown. Some force extension curves (c) displayed a characteristic small (20 nm) plateau (marked with a green arrow) that appeared after the first nonspecific binding peak but preceded unfolding events. The appearance of this plateau corresponds well to SMD predicted small pre-burst extension of BEB domains.

Figure 8a,b shows representative force spectra. A WLC model fitting was performed (with a persistence length of 0.4 nm) to determine increments in the contour length (L_c_) due to a domain unfolding. The values of L_c_ obtained (Fig. 8c) were in general agreement with SMD simulations as in curves where the protein aggregation was not seen, ΔL did not exceed 120 nm. However, the majority of measured values are below this limit, with major peaks in ΔL histograms at 20 nm, 40 nm and 60 nm (Fig. S6a). We hypothesize that this is a direct consequence of dimer detection. A rotation around the disulfide bond connecting both fragments leads to various possible configurations of this dimer during stretching as shown in Fig. S6b. Depending on the tip and the surface arrangement, several variants of standard force spectra are possible. In every case, the two bound BEB5 modules will be unfolded only partially upon AFM pulling and only a 20 nm increase in contour length from each such clamped domain can be expected. Another consequence of the expected dimer arrangement is that the BEB6 modules will not be unfolded if the pulling is made through BEB3 type modules. Additionally, the dimer arrangement significantly increases the possibility for partial or full parallel stretching of BEB3-6. When proteins are stretched in parallel, the measured increase in the contour length (ΔL) is only half of the value resulting from a single molecule pulling and twice the number of unfolding peaks can be observed^56^.

## Conclusions

Reelin is a large multi-domain protein with important roles in brain development, neuronal activity, signal transduction, cell adhesion and some types of cancer^9–11,57–59^. The characteristic BNR parts of reelin modules are well conserved among bacteria^60^ and numerous animals. So far, the interpretation of RELN role in signaling was based on the interactions of its central part with two receptors VLDLR and ApoER2. Given the role of RELN in fast growing tissues, the protein undergoes mechanical tensions. We have studied in detail dynamics and nanomechanics of the central part of RELN composed with BEB modules having common architecture. Based on series of 0.5 μs MD computed RMSF plots and correlation functions we found that all BEB modules share a similar pattern of rigid and flexible regions. Using the PRS method, we determined sensor and effector residues in all BEBs. These residues were found in “strategic” regions of reelin type modules. These regions are related to known signaling functions and a hypothetical proteolytic function. Both PRS sensor/effector residues and the correlations maps allow for better understanding of chemical signal relay pathways.

The AFM force spectroscopy study of the same central fragment of RELN was only partially successful. The protein tends to aggregate in a wide range of conditions tested, and is at least partially in a cysteine bridge-linked dimeric form. Nevertheless, a limited number of successful force spectra corroborate main features of SMD calculated mechanical unfolding scenarios of BEB modules and support the presence of intermediate pre-burst state. We have found that BEB modules, due to their unique architecture, abundant in disulfide bridges, are resistant to mild external force distortions including up to 2 nm long extensions. The forces of the order of 60 pN are sufficient to partially unfold individual BEB modules. Moreover, the unfolding simulations revealed the importance of SS_0_ disulfide bonds in BEB6 modules protecting hypothetical RELN enzymatic function. The data accumulated in this study provide background for further rational mutational studies of RELN and other proteins rich in reelin-type architecture modules.

## Supplementary information

Supplementary Material

Dataset 1

## Data Availability

The datasets generated during and/or analyzed during the current study are available from the corresponding author on reasonable request.
