# Supplementary material for: Dynamics, nanomechanics and signal transduction in reelin repeats
Source: Sci Rep. 2019 Dec 12;9:18974. doi: 10.1038/s41598-019-55461-8 (PMC6908669; doi:10.1038/s41598-019-55461-8)
Supplement: Supplementary file 1 — Supplementary Material [file 41598_2019_55461_MOESM1_ESM.pdf]

Supplementary materials for

Dynamics, nanomechanics and signal transduction in reelin repeats

Karolina Mikulska-Ruminska*, Janusz Strzelecki, Wieslaw Nowak*

Institute of Physics, Faculty of Physics, Astronomy and Informatics, Nicolaus Copernicus University, Grudziadzka 5, 87-100 Torun, Poland

*Corresponding authors: karolamik@fizyka.umk.pl (K. Mikulska-Ruminska), wiesiek@fizyka.umk.pl (W. Nowak)

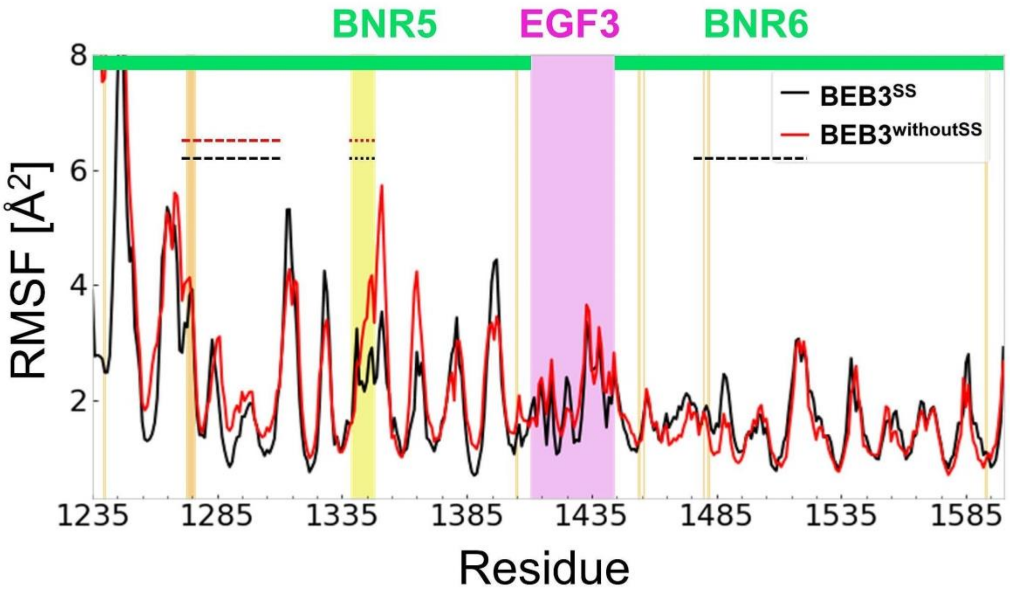

Figure S1. Comparison of RMSF values for BEB3 with and without SS_2 disulfide bond in BNR6 domain colored in *black* and *red*, respectively. Results are calculated as averages for C_α atoms based on the last 90 ns of five 100 ns MD simulations. Higher fluctuations are present in BEB3 without SS_2 disulfide bond in BNR6 domain. BNR domains are denoted by *green bars* and the regions that belong to EGF domains are in *pink*. *Yellow bars* highlight the loop that is protected by the SS_3 disulfide bond. *Orange* sites are residues within 3.5 Å from Ca^{2+} ions (see **Table S1**). *Red* and *black dotted lines* along the abscissa highlight regions that are connected by the disulfide bonds.

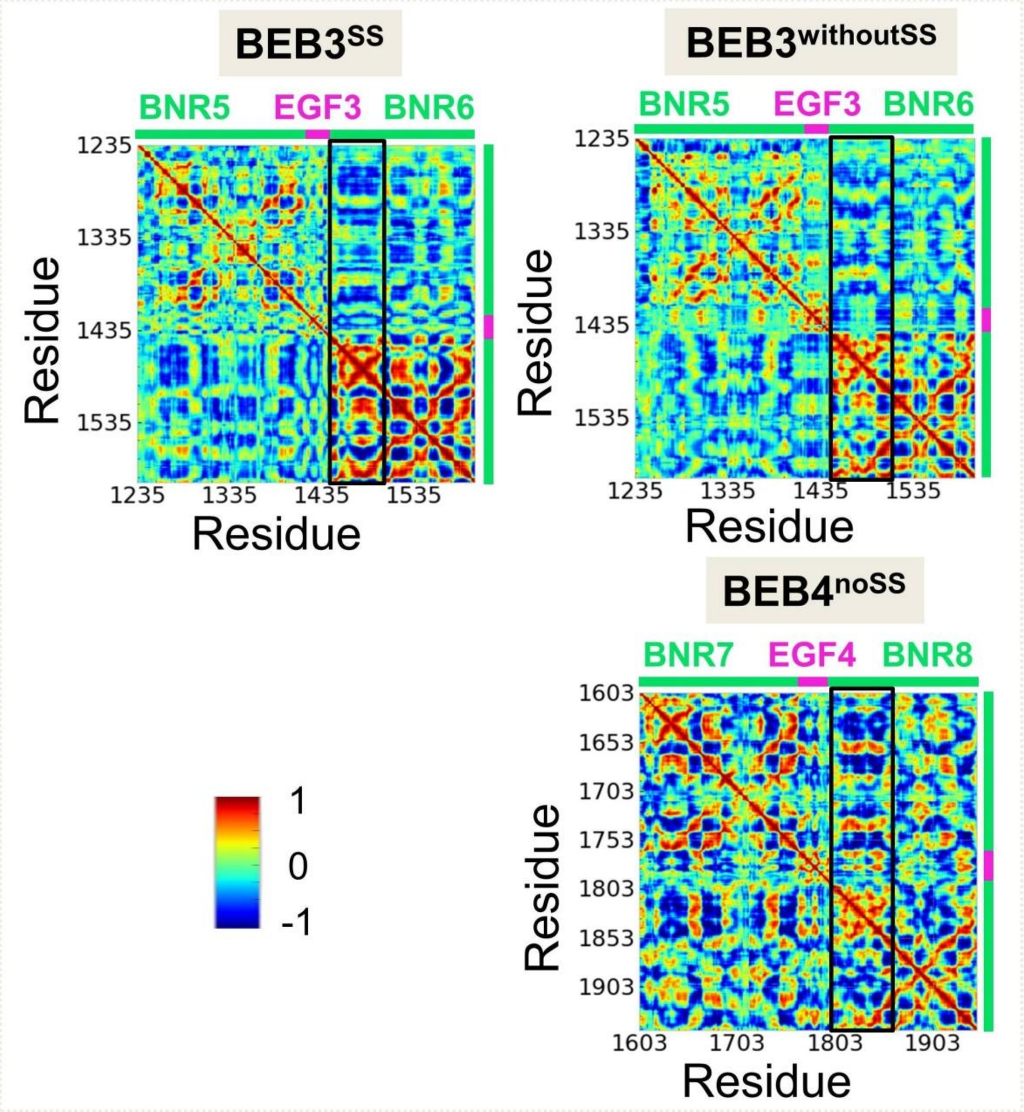

Figure S2. Cross-correlation of BEB structures in MD simulations. Cross-correlation heatmaps for individual BEB structures: BEB3, BEB4 and BEB3 without SS_2 disulfide bridge (C1475-C1522) are showing which regions tend to move in the same (correlated, colored in *red*) or in the opposite (anti-correlated, colored in *blue*) directions with respect to each other in the principal modes. *Black boxes* mark the first half of the BNR-B domain, which contains regions slightly more anticorrelated (*blue*) and less correlated (*red*) after deleting the SS_2 disulfide bond in BNR-B domain of BEB3 module. The pattern of internal correlation in BNR6 unit after SS_2 disulfide bond removal resembles that of BNR8.

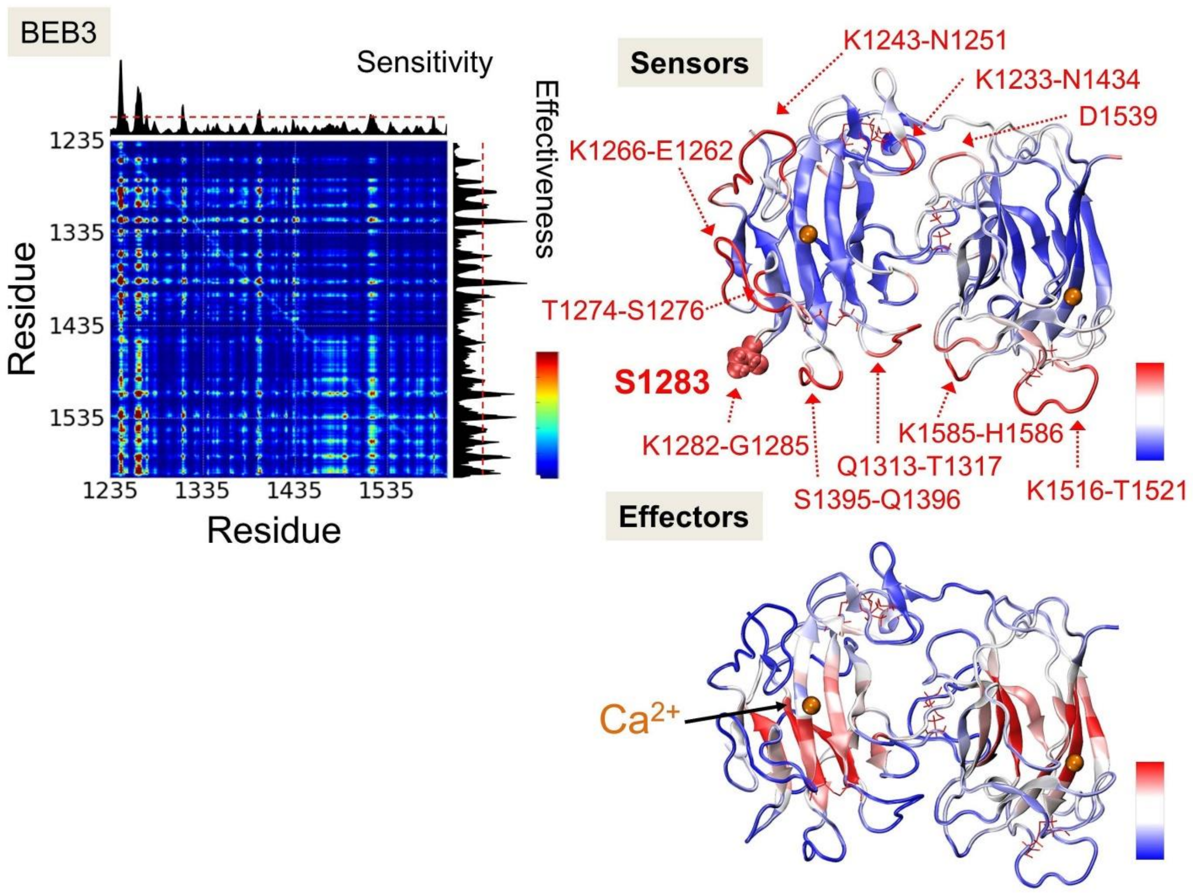

Figure S3. PRS analysis of BEB3 module. PRS map illustrating the strongest perturbation (in *dark red*) which describes the effect of perturbing residue *i* on the dynamics of residue *j.* Sensitivity and effectiveness profiles are shown. The highest values on sensitivity profile correspond to the strongest sensors and the highest values of effectiveness correspond to the strongest effectors. Both profiles are displayed on the crystal structure of BEB3. Ca^{2+} ions are denoted by *orange* spheres. Residues which are strong sensors are displayed. S1283 (strong sensor) is essential for reelin's proposed serine protease activity. Disulfide bonds in the 3-D structure are displayed as *red thin sticks*.

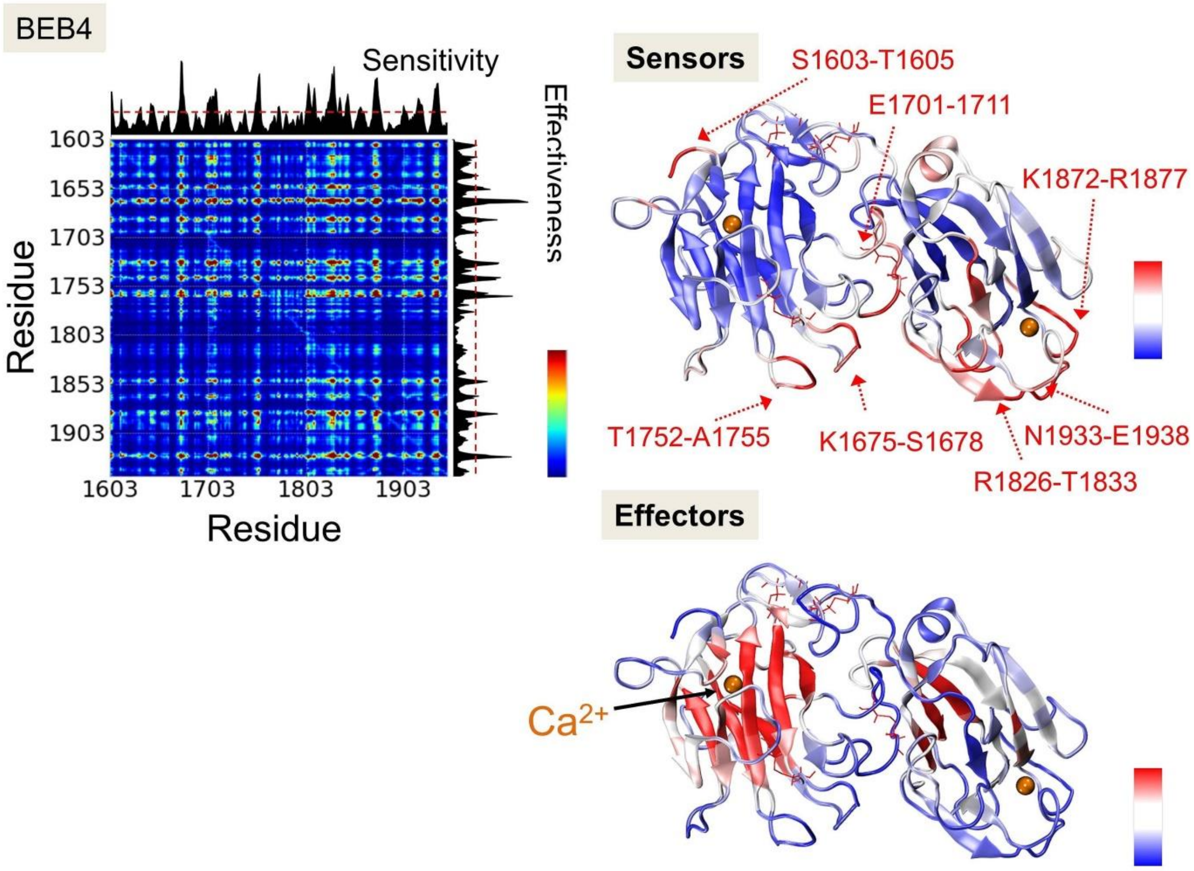

Figure S4. PRS analysis of BEB4 module. PRS map illustrating the strongest perturbation (in *dark red*) which describes the effect of perturbing residue *i* on the dynamics of residue *j.* Sensitivity and effectiveness profiles are shown. The highest values on sensitivity profile correspond to the strongest sensors and the highest values of effectiveness correspond to the strongest effectors. Both profiles are displayed on the crystal structure of BEB4. Ca^{2+} ions are denoted by *orange* spheres. Residues which are strong sensors are displayed. Disulfide bonds in the 3-D structure are displayed as *red thin sticks*.

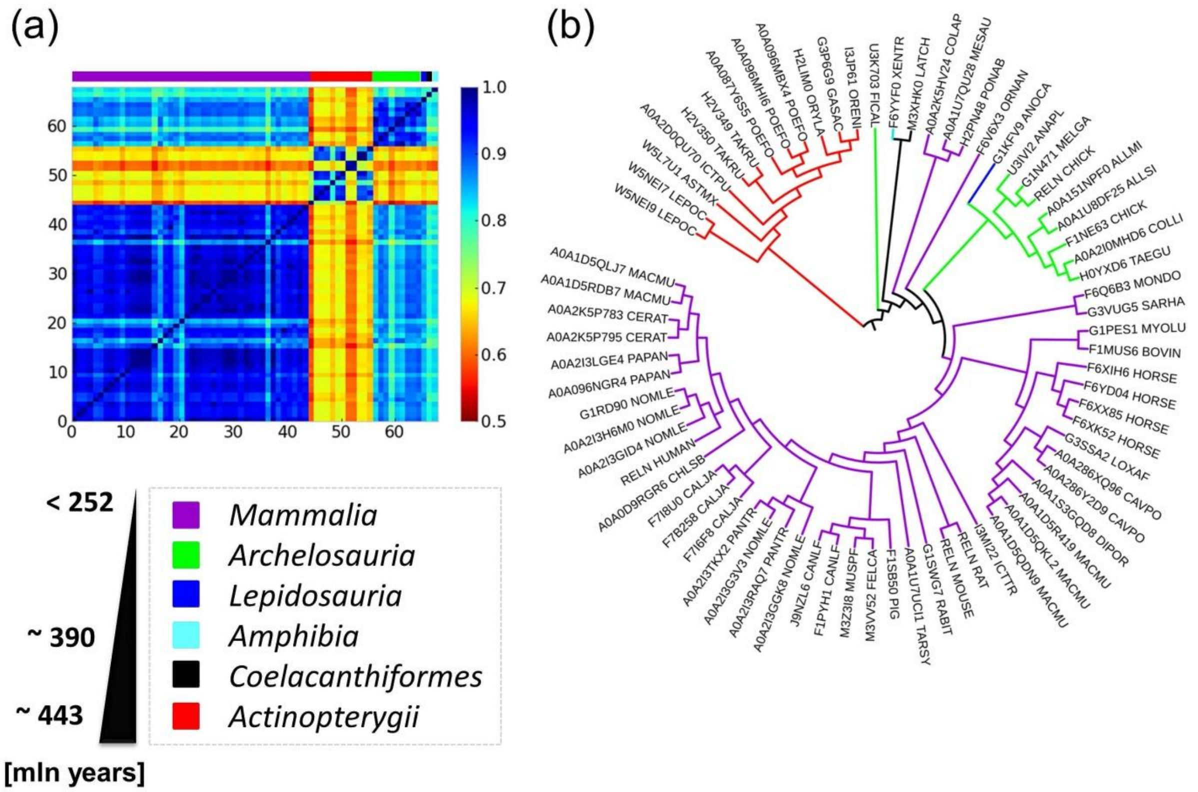

Figure S5. Comparison of different RELN sequences. The comparison of 67 sequences obtained from UniProt database is displayed as a matrix and a dendrogram. Colors of bars on the top of the matrix and colors of the branches correspond to the six classes of species, and their divergence time is displayed in the legend. The pairwise fractional sequence identity (SID) is shown in the matrix and the Hamming distance (1 – SID) is used for the sequence dendrogram.

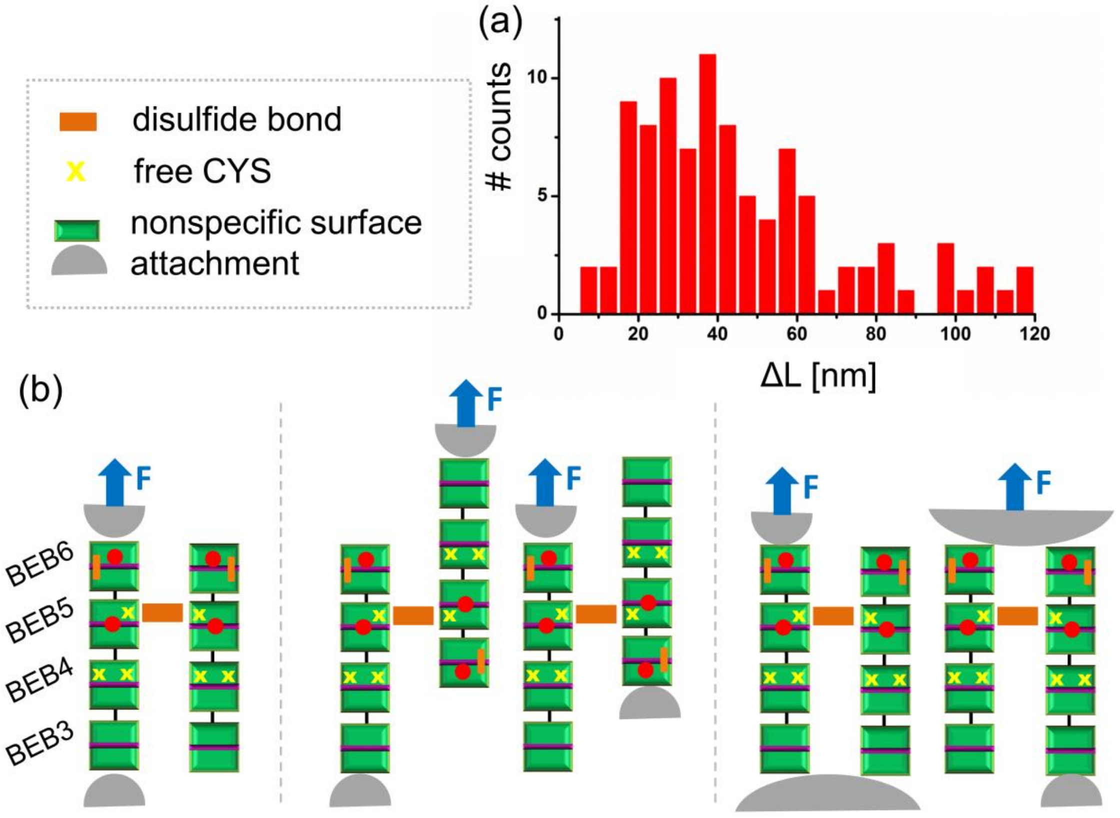

Figure S6. Distribution of contour length increments (ΔL) measured by AFM force spectroscopy for BEB3-6 RELN. The histogram of contour length increments (a) obtained with WLC model fits shows majority of values below the SMD predicted BEB3-5 structure unfolded length of ~100 nm. Dimer architecture of RELN, created with a disulfide bond between BEB5 fragments offers a possible explanation of such distribution. (b) The stretched dimer complex can lead to situations where BEB6 would not experience stretching. Additionally, the arrangements when two BEB3-5 fragments that are stretched in sequence or in parallel can also be expected. Such hypothesized complex RELN dimerization is perhaps a major cause for why smaller ΔL values dominated in AFM measurements, as compared with SMD simulations where single, individual BEB modules were unfolded.

Table S1. List of important residues in RELN structure extracted from crystal structures (within 4 Å).

Zn^{2+} interface	Ca^{2+} interface	apo-ER2 interface
BEB5: Y2060, H2061, H2074, L2261, E2264,<br>BEB6: E2397, E2399, R2458, H2460	F1240, T1273, T1274, P1275, S1276, D1405, R1454, E1456, D1480, R1482, D1593, K1610, M1636, T1638, D1761, D1808, K1835, D1943, D1944, T1961, D1963, E1993, S1995, D2125, D2173, E2175, N2202, D2310, Q2311, D2327, T2351, D2353, D2474, N2522, F2523, N2524, S2549, L2551, D2657	L1981, P1984, Y1985, S1986, S1987, L1988, G1989, P1991, E1993, Q2086, Q2118, P2153, G2154, G2176, Q2177, P2191, S2192, R2193, K2194, R2345, I2358, E2359, K2360, K2467, Q2468, T2470
